# Exploitation of endophytes for sustainable agricultural intensification

**DOI:** 10.1111/mpp.12483

**Published:** 2016-12-04

**Authors:** Kate Le Cocq, Sarah J. Gurr, Penny R. Hirsch, Tim H. Mauchline

**Affiliations:** ^1^ Rothamsted Research North Wyke Okehampton Devon EX202SB UK; ^2^ Department of Biosciences Geoffrey Pope Building, University of Exeter Exeter EX44QD UK; ^3^ Rothamsted Research West Common Harpenden Hertfordshire AL52JQ UK

**Keywords:** agriculture, bacterial endophytes, fungal endophytes, sustainable intensification

## Abstract

Intensive agriculture, which depends on unsustainable levels of agrochemical inputs, is environmentally harmful, and the expansion of these practices to meet future needs is not economically feasible. Other options should be considered to meet the global food security challenge. The plant microbiome has been linked to improved plant productivity and, in this microreview, we consider the endosphere – a subdivision of the plant microbiome. We suggest a new definition of microbial endophyte status, the need for synergy between fungal and bacterial endophyte research efforts, as well as potential strategies for endophyte application to agricultural systems.

## The Sustainable Intensification Challenge

Agricultural practices are under pressure to provide increased yields to feed the growing global population, which is expected to reach 9.7 billion by 2050 (DESA, 2015). In the period 2010–2012, 12.5% of the current world's approximate 7.6 billion population was estimated to be malnourished (FAO, 2012). Both abiotic and biotic stresses place limitations and make agricultural yields unpredictable. For example, fungal pathogens of wheat alone are estimated to cause losses of up to 29% of the crop (Oerke and Dehne, 2004), and other groups of pathogens and various abiotic challenges, such as flooding, drought and soil fertility, place further pressure on production. Moreover, climate change is predicted to increase the frequency, number of locations and severity of these threats. Focus must be directed towards sustainable intensification of agriculture under fluctuating and unpredictable conditions, as well as the minimization of the threat of pathogens and abiotic stresses.

## What is an Endophyte?

We consider the endosphere to be separate from the phylloplane, rhizosphere and rhizoplane. A range of organisms and complex interactions have been described within plant tissues. These include fungi, bacteria, viruses and fungal–bacterial symbioses. However, as highlighted by Hyde and Soytong (2008), there is a confusing series of definitions to describe an endophyte. These range from the original endophyte definition by de Bary (1866), who wrote, ‘any organisms occurring within plant tissues’, to the fungal‐centric description by Rodriguez *et al*. (2008): ‘a fungus which spends its life‐cycle within a plant only emerging and undergoing sporulation upon senescence of the plant tissue’. In addition, a wide variety of bacterial endophytes capable of growth and survival on roots and in the soil have been described. Recent reviews of bacterial and fungal endophytes suggest that the term endophyte should refer to ‘habitat only, not function’, and should include ‘all microorganisms which, for all or part of their lifetime, colonize internal plant tissues’, referring to the continuum of interaction between a host plant and the microbes that colonize it (Hardoim *et al*., 2015; Schulz and Boyle, 2005). Here, we propose an amended definition. This is similar to the definition of Hallmann *et al*. (1997), but considers all contributing microbes: ‘Endophytes are microbes which occur within plant tissue for at least part of their life cycle without causing disease under any known circumstances’. This caveat means that some microbes may be currently considered endophytic, but this designation may be changed if they are subsequently shown to be harmful to a plant host. It is interesting that the genomes of fungi that are currently perceived as endophytes often retain plant pathogenicity genes. It seems that altered gene regulation and gene disruption, rather than deletion, are important in the development of a non‐pathogenic relationship with the plant host (Hacquard *et al*., 2016; Xu *et al*., 2014). At present, there is little understanding of how the genomes of bacterial endophytes and plant pathogens differ, and this represents a knowledge gap.

## Can Endophytes Boost Crop Production?

Soil fertility in modern agricultural systems is maintained by the application of fertilizers, and pathogens and pests are controlled by various agrochemicals. Groups of microbes, such as mycorrhizal fungi and nitrogen‐fixing bacteria, have long been known to benefit plant growth (Berendsen *et al*., 2012; Santoyo *et al*., 2016). In addition, some endophytic microbes residing within plant tissues have been shown to promote plant growth and endow protection against biotic and abiotic stresses under laboratory conditions (Baltruschat *et al*., 2008; Hubbard *et al*., 2014; Waller *et al*., 2005). However, when coupled with uncontrollable variables, the protective outcome observed for endophytes under laboratory conditions can be less effective in field conditions (Serfling *et al*., 2007). In contrast, reliance on fungal endophytes to protect ryegrass against the Argentine stem weevil has driven the discovery and application of endophytes, such as AR1 and AR37, as protective agents that are also non‐toxic to livestock in New Zealand grasslands (Easton *et al*., 2001; Rodriguez *et al*., 2009; White *et al*., 2002).

## The ‘Endomicrobiome’

**Figure 1:**
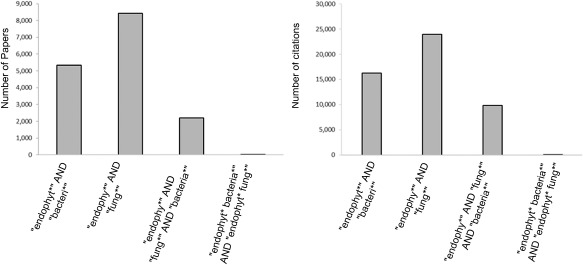
Literature search results for studies on bacterial and fungal endophytes. Web of Science search results on 24 May 2016 for terms that returned results for bacterial endophyte, fungal endophyte as well as fungal AND bacterial endophyte demonstrates the lack of coordinated research into fungal and bacterial community endophyte studies.

To date, endophyte research has focused largely on fungal or bacterial entities separately. Indeed, few researchers have considered the combined effect of the ‘endomicrobiome’. There is a clear distinction within the literature between research articles that consider both fungal and bacterial endophytes within the same work. Here, three literature search engines that are commonly used within the scientific community were interrogated for journal articles published in the last 5 years that included variations of the terms ‘fungi’, ‘bacteria’ and ‘endophyte’ (Fig. 1). The results highlighted the lack of crossover in articles that considered beneficial bacterial and fungal endophytes. Table 1 contains a full breakdown of the terms searched. The relationships and functions within endophytes themselves may be important, and it would be short sighted to study them in isolation.

**Table 1:** Number of returned hits for related searches across three commonly used literature databases.

Search term	Web of Science 2010–2015 (TOPIC)	Google Scholar 2010–2015	Scopus 2010–2015 (article title, abstract, keywords)
‘endophyte’ AND ‘fungal’ AND ‘bacterial’	518	5900	321
‘endophyte’ AND ‘fungal’	1409	12000	1833
‘endophyte’ AND ‘bacterial’	758	8480	998
‘bacterial endophyte’	68	976	235
‘fungal endophyte’	434	4120	772
‘bacterial AND fungal endophyte’	1	9	6

Search results on 24 May 2016 for terms that returned results for bacterial endophyte, fungal endophyte as well as fungal AND bacterial endophyte demonstrates the lack of coordinated research into fungal and bacterial community endophyte studies across three widely used literature databases.

**Figure 2:**
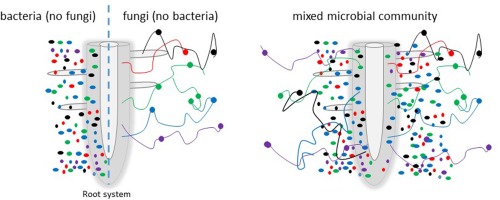
Schematic diagram demonstrating the limitations of studying fungal–plant and bacterial–plant interactions in isolation. Bacteria and fungi interact within the bulk soil, rhizosphere, rhizoplane and endosphere. Fungi and bacteria provide shared and contrasting services to the plant host. In addition, fungal networks are considerably more mobile than bacterial cells and can vector bacteria to the plant host.

Much research highlights how plants benefit from endophyte infection; however, there is also evidence that some fungal pathogens begin their lives endophytically, surviving within the plant tissue without causing symptoms and without known detection by the plant (Carroll, 1988). The mechanisms by which this switch in microbial lifestyle occurs is crucial to understanding the balance between beneficial and pathogenic plant–microbial relationships (Saikkonen *et al*., 1998; Schulz and Boyle, 2005). This may be influenced by many environmental factors, which may have varying levels of importance during different stages of the plant and microbial life cycle, depending on the conditions. The mode of entry to the plant is an important factor to consider, as microbes can be transmitted vertically, through seeds. This could be an indicator of their pathogenic potential. In addition, both fungi and bacteria can enter through stomata and be transferred horizontally from plant to plant (Hardoim *et al*., 2015). Bacteria can theoretically use fungal hyphae as vectors and can be transferred to the host by this route, which highlights the importance of a holistic approach in plant–endophyte studies (Fig. 2), and the concept of the mycosphere, where fungal surfaces provide a niche for bacterial growth, is receiving more attention (Haq *et al*., 2014). There are a few well‐known examples of fungal endobacteria (e.g. Moebius *et al*., 2014), some of which infect mycorrhizal fungi (e.g. Torres‐Cortés *et al*., 2015), but when considering that the vast majority of microbial biomass in soil is derived from bacteria and fungi, there is only a small amount of co‐ordinated research between these Kingdoms (Table 1).

It has often been assumed that endosphere colonization is a passive process. However, Robinson *et al*. (2016) demonstrated that the abundant rhizosphere bacterium *Bacillus mycoides* was unable to colonize the endosphere of wheat in a gnotobiotic system in the absence of competing bacteria, suggesting that colonization is gated. It will be interesting to determine what proportion of the rhizosphere/rhizoplane community can actually become endophytic.

## Culture of Endophytes

Numerous papers have described various methods for the culture of endophytic organisms. They involve the surface sterilization of plant tissues (to various degrees), maceration or disruption, and then plating the tissues onto a medium which supports growth of the organisms. More sporadic within the literature are descriptions of controls undertaken to ensure that the outer tissue is indeed free of viable microorganisms. It is clear that plant tissue surface sterilization should be tailored to the tissue sample type. Here, we propose that the total final surface wash from the plant tissue section should be concentrated and cultured to determine the absence of viable organisms (Robinson *et al*., 2015). In addition, isolated organisms should be referred to as putative endophytes unless they have been positively identified within the host using microscopy or passaged through the host, as demonstrated in wheat by Robinson *et al*. (2016).

Until recently, only 1% of microbes present in bulk soil have been amenable to culture. However, the development of an isolation chip, the ‘ichip’ (Nichols *et al*., 2010), has resulted in the culture of up to 50% of the microbes present in soil. It is likely that rhizosphere and endosphere colonizers are more amenable to culture than are fastidious soil organisms, as the vast majority are fast growing and respond well to nutrients, such as sugars and amino acids. The high‐diversity bacterial culture collection of Bai *et al*. (2015) seemingly supports this. It follows that ichip technology, in addition to the methods deployed by Bai *et al*. (2015), could be used to facilitate the culture of the plant‐associated microbiome.

## The Application of Endophytes to Agriculture

The best strategy for the application of endophytes in agricultural systems is not yet known. The most obvious approach is to add inoculants to the soil or as seed dressings. There are reports of this approach being successful for sugar cane (e.g. Silva *et al*., 2012). However, the use of inoculations is often unsuccessful on a field scale because of problems with the establishment of the biological agent (O'Callaghan, 2016). This is compounded by the enduring need to inoculate crops at each planting time, if vertical transmission into the seed of the microbial agent does not occur. In wheat, it was found that true vertical transmission did not occur when surface‐sterile excised embryos were incubated with potential endophytes (Robinson *et al*., 2016). Therefore, it is highly likely that seed‐adhering microbes are able to colonize the endosphere after germination, supporting the application of potential endophytes as seed dressings. An alternative approach is to amend the agricultural system to encourage the indigenous community to respond and aid host plant growth and defence, although this requires a better understanding of the soil microbiome. The high rates of inorganic fertilizers currently added to crops circumvent the need for a healthy microbiome to aid nutrient acquisition, and so it follows that lower fertilizer rates will enable the selection of enhanced beneficial interactions with endophytes. A key consideration for the introduction of endophytes is their behaviour under a range of conditions, and it is critical to understand their full life cycles and genome plasticity in order to assess their risk of becoming pathogenic, either through a shift in abiotic conditions or adaptation to an alternative host (Redman *et al*., 2001). A novel approach would be to modify the root exudation chemistry of crops to select a more beneficial microbiome – this may also be one of the factors determining cultivar responses to drought, starvation and disease.

Despite the success of a few well‐known endophyte–plant relationships (Hardoim *et al*., 2015), the use of endophytes to overcome threats to plant health is not commonplace in most conventional agriculture, and our reliance on agrochemicals continues to take precedence over alternative solutions. Currently, our widespread reliance on fungicides may incapacitate fungal biological agents (as well as the vectoring of bacterial agents by fungi), and high fertilizer levels reduce plant dependence on both fungal and bacterial endophytes, and other parts of the root microbiome. In addition, some endophyte traits may have mixed benefits from an anthropogenic perspective, e.g. *Epichloë*, which protects grasses against root‐grazing nematodes, may be toxic to vertebrates (Schardl *et al*., 2004). Indeed, any bacterial and fungal endophytes that suppress herbivory or plant diseases must be rigorously tested for toxin production for human and animal safety. We need a much better understanding of the interactions between the host and the soil microbiome in order to exploit it and recruit beneficial endophytes, as well as the interactions that take place between microorganisms in this system. This provides an imperative to consider bacterial and fungal endophytes (and, indeed, archaeal and non‐fungal eukaryotic endophytes) as part of the same system.

## Concluding Remarks

Several important questions remain unanswered concerning the practical use of endophyte ‘supplements’ in agriculture. However, with the correct management, they hold potential for the control of current and emerging pathogens, as well as biotic stresses, as we encounter deviation in these through climate change (Howden *et al*., 2007; Johnson *et al*., 2013). This is likely to be achieved through a better understanding of signalling between the host plant and the microbiome, and, ultimately, the manipulation of root exudation profiles to recruit a more beneficial root microbiome, of which the endosphere is an integral part.
